# Stakeholders’ views on an institutional dashboard with metrics for responsible research

**DOI:** 10.1371/journal.pone.0269492

**Published:** 2022-06-24

**Authors:** Tamarinde L. Haven, Martin R. Holst, Daniel Strech

**Affiliations:** 1 Berlin Institute of Health at Charité–Universitätsmedizin Berlin, QUEST Center for Responsible Research, Berlin, Germany; 2 Medizinische Hochschule Hannover, Institut für Ethik, Geschichte und Philosophie der Medizin, Hannover, Germany; University of Rennes 1, FRANCE

## Abstract

Concerns about research waste have fueled debate about incentivizing individual researchers and research institutions to conduct responsible research. We showed stakeholders a proof-of-principle dashboard with quantitative metrics of responsible research practices at University Medical Centers (UMCs). Our research question was: What are stakeholders’ views on a dashboard that displays the adoption of responsible research practices on a UMC-level? We recruited stakeholders (UMC leadership, support staff, funders, and experts in responsible research) to participate in online interviews. We applied content analysis to understand what stakeholders considered the strengths, weaknesses, opportunities, and threats of the dashboard and its metrics. Twenty-eight international stakeholders participated in online interviews. Stakeholders considered the dashboard helpful in providing a baseline before designing interventions and appreciated the focus on concrete behaviors. Main weaknesses concerned the lack of an overall narrative justifying the choice of metrics. Stakeholders hoped the dashboard would be supplemented with other metrics in the future but feared that making the dashboard public might put UMCs in a bad light. Our findings furthermore suggest a need for discussion with stakeholders to develop an overarching framework for responsible research evaluation and to get research institutions on board.

## Introduction

Concerns about research waste have fueled debate about incentivizing individual researchers and research performing organizations to conduct responsible research [1]. One key point was that individual and institutional research performance should be assessed differently [2–4]. Rather than looking at high impact publications or grants obtained, one should focus on indicators that pertain to responsible conduct of research [5–7]. This holds true for individual researchers of all career stages [8], as well as for research performing organizations that can themselves be subject to dysfunctional incentives [9, 10].

Research performing organizations, in our case, University Medical Centers (UMCs), play a key role in fostering responsible research [11–13]. They can put out relevant policies (e.g., for data sharing), provide critical infrastructure (e.g., for coordinating clinical trials), and reward responsible research more generally [14]. But this requires awareness about responsible research on an institutional level, and commitment from UMCs to make this a priority [15, 16].

In this study, we showed stakeholders a proof-of-principle dashboard with quantitative metrics that visualized responsible research performance on a UMC level (e.g., for a given UMC, what percentage of its trials is prospectively registered?). The metrics included pertained to responsible research practices such as registration and reporting of clinical trials (inspired by work from Goldacre et al. and Wieschowski et al., [17, 18]), robustness in animal research (e.g., randomization and blinding, see Macleod & Mohan [19]), and Open Science (including Open Access, Data, and Code, see Serghiou et al. [20]). The goal of our study was twofold: first, to explore stakeholders’ views on the dashboard and its metrics and second, to use their feedback to improve the proof-of-principle dashboard before making it public (not described in this report). Our research question was: What are stakeholders’ views on a dashboard that displays the adoption of responsible research practices on a UMC-level?

## Materials and methods

### Ethical approval

The ethical review board of the Charité–Universitätsmedizin Berlin reviewed and approved our research protocol and materials (#EA1/061/21).

### Participants

When discussing the adoption of responsible research practices on an institutional (UMC) level, we distinguish four main stakeholder groups: 1) UMC leadership (e.g., deans, heads of strategy, heads of pan-UMC organizations), 2) support staff (e.g., policy makers tasked with topics such as Open Science (“Open Science Coordinator”, “Innovation Manager Open Science”), librarians, especially those working on Open Access (“Open Access Coordinator” or bibliometrics expert), heads of core facilities (including 3R centers and clinical research units)), 3) research funders, and 4) experts in responsible research assessment. We chose these groups, because they represent parties that might have a say in whether the approach gets adopted for a particular UMC (group 1), how it could get adopted (group 2), whether the adoption will be encouraged (group 3), and whether the proposed approach is in line with ongoing ideas to promote responsible research (group 4).

#### Recruitment

We used purposive sampling where individuals were invited based on a specific criterion, namely their role or alleged specific expertise associated with the stakeholder group. This is a sampling technique intended to identify individuals that are assumed to be representatives of their role and that can provide detailed information. However, the concern with this criterion approach is that the expertise is assumed and that based on a role description, one may fail to identify those who are most knowledgeable or those with the greatest level of expertise [21]. We tried to alleviate this concern by first inviting individuals that the first and last author knew to be knowledgeable through their participation in conferences, seminars, and workshops over the last five years. Where this regarded stakeholders that the authors have worked with, or are currently working with, we denote this with an asterisk (“*”). After each interview, we asked the interviewee for recommendations of other stakeholders with specific expertise, an approach called snowballing. Finally, we also invited individuals based on internet-searches (cold calling). In total, we invited 49 individuals to participate.

#### Procedure

Participants first received an invitational email with a link to the information letter, informed consent form, and protocol (see S1 File). We sent one reminder a week after. When participants agreed to participate, we scheduled an appointment for an online interview through Microsoft Teams. Participants received a link to the dashboard and a tutorial explaining the dashboard (see here and S2 File). They signed the informed consent form prior to the interview.

We conducted the interviews online between April and June 2021. Interviews lasted between 30 and 50 minutes. One team member led the interview (TH, female (Dr), MH, male (PhD candidate) or DS, male (Prof), who are all trained in qualitative research methods and familiar with the responsible research literature), whilst another team member observed and made notes on the interview’s process and its content. After a brief introduction of the interviewer and the interviewee, interviews were conducted using a topic guide (semi-structured) that was based on a literature review and various internal discussions (see S3 File). The topic guide was pilot tested (*n* = 3) for comprehensibility using cognitive interviewing [22]. After each interview, the team got together for reflection (peer-debriefing). If they wanted, interviewees received a brief written summary of the interview (member check) with the option to comment or correct. The interviews were transcribed by a transcription company under a data processing agreement. We used the COREQ checklist to write up our findings [23]. For a more elaborate description of our procedure, see study protocol (https://osf.io/z7bsg/).

#### Data analysis

The aim of our study is exploratory and descriptive. To get a broad understanding of stakeholders’ views, we applied content analysis that involved deductive and inductive elements [24]. We structured stakeholders’ views by looking for Strengths, Weaknesses, Opportunities, and Threats (SWOTs), see Table 1. Subcodes within each part of the SWOT were derived from the data. These subcodes pertain to the general approach of an institutional dashboard and responsible metrics, i.e., suggestions for improvement by interviewees that were specific to the proof-of-principle version are not featured in the results where they have been implemented successfully (e.g., the dashboard interviewees saw displayed a sample of publications, the final dashboard that will be published shortly includes all publications).

**Table 1:** SWOT definitions.

**Strengths**	Characteristics inherent to the current dashboard approach or the metrics included that are considered valuable for visualizing institutional performance in terms of responsible research
**Weaknesses**	Characteristics inherent to the current approach that could be considered disadvantages, or areas that need improvement for the dashboard and that are within the realm of the internal environment, meaning the creators of the dashboard could undertake action to alleviate these concerns
**Opportunities**	Potential use cases of the dashboard that could increase its chances of successful uptake, as well as additions to the approach or the metrics that–when implemented–would improve the chances of stakeholder wide-uptake of visualizing institutional performance in terms of responsible research
**Threats**	Characteristics in the external environment that could undermine implementation of the dashboard approach, or others measures, to visualize institutional performance in terms of responsible research

Our analysis consisted of three phases. First, two team members (TH and MH) read and coded 5 interviews independently using MAXQDA 2020 (Release 20.3.0, VERBI GmbH, Germany). They then each proposed a code tree, exchanged these, and resolved discrepancies (mostly whether something was best classified as a strength or an opportunity, a weakness or a threat) through discussion. With this revised code tree, the two team members coded another 5 interviews. This process was repeated until saturation was established [25], meaning that when coding additional interviews, no new codes were identified. The code tree that resulted was then presented to the full research team (together with the interviews). We analyzed all interviews. Yet, reviewing the final three interviews resulted in minor modifications to the wording of the code tree only (SI 4), which formed the basis of our results.

## Results

### Demographics

We invited a total of 49 stakeholders, 28 of them agreed to an interview (response rate: 57%, one interview was conducted with two participants). From these 28 stakeholders, 89% were identified through the criterion approach, 11% were suggested through snowballing, and 0% of individuals from internet search contacts participated. The demographics of our interviewees appear in Table 2.

**Table 2:** Demographics of interview participants.

Stakeholder group	#
*UMC leadership*	4
*Support staff (including librarians*, *policy makers*, *CRU staff*, *3Rs staff)*	14
*Funders*	5
*Responsible research experts*	5 (* = 3)
**Gender**	
*Male*	22
*Female*	6
**Nationality**	
*Germany* ^	17
*Europe (besides Germany)*	10
*North America*	1
**Total**	28

**^** One interview was conducted in German.

* The authors have previously collaborated with 3 experts in responsible research.

**Fig 1:**
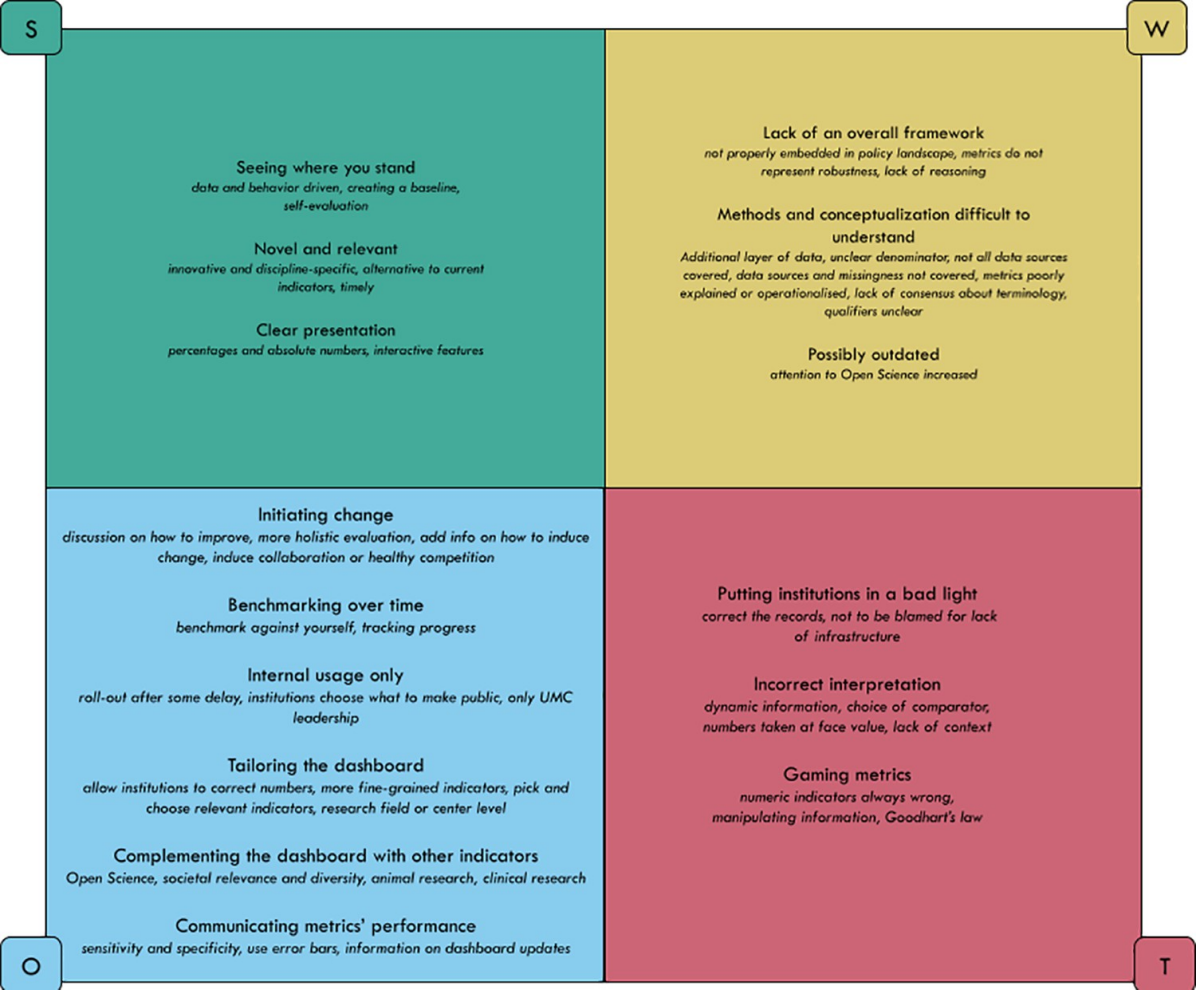
Overview of SWOTs regarding institutional dashboards with metrics for responsible research.

Below we describe the most important SWOTs according to our interviewees. We identified 3 strengths, 3 main weaknesses, 6 opportunities, and 3 main threats, see Fig 1. Each subtheme is illustrated with quotes, see Table 3.

**Table 3:** Illustrative quotes per theme.

SWOT category	#	Illustrative quotations
***Strengths ***		
Seeing where you stand	1	“You need to be a little tough, to be honest to yourself and see where you stand. It might be uncomfortable at the beginning when you think, "all right, we are the [UMC], we are the best, the nicest and the greatest.” (Interviewee 19 –Support staff 3R)
	2	“You can’t change unless you know what your baseline is.” (Interviewee 4 –Responsible Research expert)
	3	“If we as a funder ask for these things, are we actually in line with the strategy of the institution we are planning to fund, or is this really something we would be imposing, or is this something we are behind? It’s really hard to tell how far the institutions are, because in applications, they will always tell you, "Everything is fine. We have this policy, blah blah blah." But to actually see the numbers really helps. And this, I find really interesting.” Interviewee 13 –funder)
Novel and relevant	4	“Some reviewers maybe don’t take the time for it or they look for the h-factor or the impact factor, sorry, by themselves even though you don’t want them to do. This is really a problem and this is why I think we don’t really do this at the moment but I think it could be very interesting to have alternative parameters, not only say look into the papers by yourself, but we have indicators like randomization, blinding, and power calculation for the reviewers.” (Interviewee 14 –funder)
	5	“It’s a positive view. I’m feeling very positive about this because it demonstrates the value of openness and also the meaning of openness and scholarly communication. I think it makes researchers, but also administrators, aware that open access and open science practices matter and that techniques are available to monitoring the progress of these open access and open science practices.” (Interviewee 24 –Responsible research expert)
Clear presentation	6	“I like the simplicity of the dashboard, so that you have these indicators and that the context is given when you hover over these information or warning signs with limitations. I really, really like the limitations button so that people know how to interpret the data. So, there’s a nice balance between simplicity and context. I also like the fact that there is the percentage and the absolute number because often, yes, one of the two is given” (Interviewee 22 –librarian and Open Science expert)
	7	“The idea of the dashboard obviously seems to be that you have a quick overview and I don’t have to go into many details there but that was my idea of a dashboard and in terms of that I think it’s quite helpful.” (Interviewee 20 –UMC leadership)
***Weaknesses ***		
Lack of an overall framework	8	“I don’t see a conceptual scheme behind it yet. So, what do you want to measure? You seem to jump immediately to what you can measure. That is one of the things I was missing, jumping immediately in the doable, and I didn’t see the analysis of what you ideally would want to do. […] That type of reasoning, I was missing. It was jumping to what can be automated, jumping to what is available, jumping to what was out, that not that many effort can be made graphs of.” (Interviewee 7 –Responsible research expert)
	9	“There is a critical point that came into my mind, and that is the question, who says which and who gives the standard? So, who says that these metrics are the right ones? And who says that the way they were calculated are correct? So, there must be a really good justification that these metrics are correct and they should be something like a general agreement.” (Interviewee 19 –Animal research expert)
Methods and conceptualization difficult to understand	10	“Then, I would also have, when I would be a dean of a UMC, I also should be able to defend the whole thing, to be realistic, because you’re running a shop of researchers. They immediately start criticizing the methodology behind the whole thing. So the methodology should be completely transparent. Well, it will never be excellent, but it should be acceptable, and good enough for purpose. Fit for purpose. I should be able to defend that fiercely, because I need to defend that, as a leader of such an institution.” (Interviewee 7 –Responsible research expert)
	11	“Then listed are three measures against the risks of bias, like randomization, blinding, sample size calculation. They are important measures. I agree, but I don’t think that are comprehensive enough to qualify as indicators of robustness, it needs more than that, I think.” (Interviewee 28 –Support staff; Animal research expert)
Possibly outdated	12	“You see the open access dashboards and you see it over time, you know, but 2018 is nice, but first of all, I would challenge you to make dashboards as accurate as possible because open access is moving so rapidly that 2018, yes well, if there is one big Springer deal in place after 2018 or Elsevier deal in place after that, or there’s no deal, then the numbers drop or improve so that’s my first point make it as accurate as possible.” (Interviewee 1 –Librarian and Open Access expert)
***Opportunities ***		
Initiating change	13	“If you’re looking at it like if you want to improve open science and robust research, then it’s a good thing because if you see the average and you see that your own institution is below average, then that gives you an indication that something should be done or it gives you an idea that maybe you should ask questions, why my institution is below average. And that’s good. Then you can start to talk to people and do something about it, try to find reasons for this” (Interviewee 12 –Librarian and Open Science expert)
	14	“So they become a regular, because they are so aggregated and so information-rich, they should be on that level and really a general part of the discussion on a regular theme, because this could also foster the discussion in this area.” (Interviewee 26 –science management and strategy)
Benchmarking over time	15	“Because looking at what happens over time within a center, that to me is probably the most informative of this whole thing. I’m not so keen on ranking UMCs. I’m not so keen on doing races between institutions, that is rather trivial. What you want is their progress in the right direction. You want to be able to pick that up, and that you should do on center level and on indicator level.” (Interviewee 7 –Responsible Research expert)
	16	“Where I’m currently is like an absolute information that doesn’t give me much discussion points. So it would be helpful for me if I could decide, what to change over time, and maybe if I, as a board member, had made a decision two years ago and then I would like to see what changes.” (Interviewee 26 – science management and strategy)
Internal usage only	17	“So for me, the strength really lay in considering this, for example, as an instrument of self-analysis. That is, if it remains at the level and is not linked to "I’ll show this at the next review and then my bar will be higher than that of the others", but if it is a kind of internal process analysis or an integrated part of an internal process analysis.” (Interviewee 8 & 9 –funder)
	18	“The question is, the moment you go public, we have the blaming issue and then the press comes in and then it’s hard to get better. So it’s not a question of "do we have a benchmark"? It’s a question of at least the first two or three years, can we keep the data in a protected space where people from different institutions can deal with it very open minded and discuss why they think the differences are there.” (Interviewee 23 –UMC leadership)
Tailoring the dashboard	19	”Some institutions may be particularly interested in certain things and others may be interested in very different things. But I think we need to get them to talk about other, a handful or three core practices that we could work on across the board.” (Interviewee 4 – Responsible Research expert)
	20	“I think not only in the medical sciences but especially there because it’s so it’s a very broad field. And I know I know it from the discussion about the impact factors that are very heterogeneous with regard to the disciplines. Whether we have a very, let’s say, visible discipline, like, I don’t know, cardiology or something, and then you have like the small disciplines, like ophthalmology. It’s very small disciplines and with I think very strong effects on the impact factors. So this could be interesting when looking at your robustness indicators to have a differentiation by the disciplines.” (Interviewee 14 – funder)
Complementing the dashboard with other indicators	21	“An indicator that I missed actually was, whether there’s a pre-print or not. PubMed Central. We invested a lot the last two years to link pre-prints with the accepted version and the journal, and this could be really of added value. Also to demonstrate to researchers and decision makers that, pre-printing can matter and can lead to quality-assured publications.” (Interviewee 24 –librarian and Open Science expert)
	22	“I think for transparency, you have also to be clear where are conflicts of interest?” (Interviewee 15 –funder)
	23	“The problem is to not focus only on those things that are easily measured, like open data, number of publications, but also like the more soft "how" things. And they can be expressed also. Like inclusiveness and diversity can be expressed extremely easily in this way. I tell you, that will be scary for <UMC>.” (Interviewee 10 –Responsible Research Expert)
	24	"You could ask whether stakeholder or patient advocacy groups, for example, were involved in a trial design and preparation of the trial, and perhaps trial conduct or so. But as I said, the patient perspective on how patients who were in a given trial, how they viewed the overall conduct of the trial, I think is another aspect that might be valuable at some stage.” (Interviewee 27 –UMC leadership)
Communication of metrics’ performance	25	“[A]s a methodologist maybe, you need to show me what is the validity and the precision of the whole game. Validity in terms of sensitivity, specificity, predictive values, what have you, and precision in terms of test sample and confidence intervals, and whatever. There is no confidence interval in all these neat graphs, which is worrying, because you seem to suggest that there are differences, and I’m not convinced. You might only be looking at random fluctuation” (Interviewee 7 –Responsible Research expert)
	26	“I think the metrics that you have are fine, but I’m not sure if they are accurate. So it depends on the quality of the data you use to produce the dashboard. So I can’t tell what the quality of your data input is. You should make sure that the data you show are reliable” (Interviewee 12 –Responsible Research expert)
***Threats ***		
Putting institutions in a bad light	27	“[I]t’s not really important what others do, we try to achieve 100 percent, that’s not easy, but we would be working on this now. For us, it’s important to set goals from the beginning and to achieve those goals. Yes, it’s always the wrong way to get bad press and then you investigate, what’s the reason for that? And then you gain goals from the bad press. The better thing is to set from the beginning, what are our goals despite of all others and of the press and them to achieve this?” (Interviewee 16 –clinical trials expert)
	28	“I think people worry that this information will be used to point fingers in a negative way. And I think we as a community must work very, very hard, collaboratively with the end users to improve the situation.” (Interviewee 4 –Responsible research expert)
Incorrect interpretation	29	“[T]his is a very tricky part because, once a civil servant of a university that I worked for said, "Please keep in mind that you can make a very nice report with an executive summary, but these people, these policymakers, they are like children. They read comic books. So they only look at your tables and graphs, and everything else, the text, is simply overseen, overlooked or forgotten." So you can put a lot of effort in writing down extensive sections with limitations, but not so many people will read these, which is, of course, stupid. I realize that and I’m aware of that. But what can you do about it?” (Interviewee 6 –Open Science expert)
	30	“I think I do, but in the conversations that we’ve had with university leadership, we noticed that a lot of people don’t. They just look at the figures and say, "oh, okay", they take it at face value. And don’t really appreciate the limitations that are that are there.” (Interviewee 5 –Librarian)
Gaming metrics	31	“There we are again at the end with ’Goodhart’s Law’. If at some point this becomes a measure that is perhaps linked to success in acquiring third-party funding, then everyone will end up writing in: ‘I share my data with. . .’ How reliable this is then the second level? But these numbers can increase rapidly if you only link them to an output link at the end. In this respect, it may seem objective now, but in the end it is no longer objective when you put it in front of the cart.” (Interviewee 8 & 9 –funder)
	32	“I think that all the potential dangers of a dashboard are always that the metrics are going to be taken as a goal and perhaps also that it’s going to be seen more as a leader board than as a way to help people move forward.” (Interviewee 3 –librarian)

### Strengths

#### Seeing where you stand

Overall, interviewees were pleased to see that the dashboard focused on specific measures that relate to concrete behaviors. Many interviewees thought the dashboard was helpful in showing institutions where they stand when it comes to responsible research practices. The dashboard approach allowed for creating a baseline, which was considered essential before talking about change or improvement on the metrics. Some expressed curiosity about the extent to which the dashboard would match internal data on self-evaluation processes.

#### Novel and relevant

Various interviewees indicated this was the first time they saw such a dashboard. They thought it fulfilled an important need and some interviewees felt these measures could serve as an alternative to publication output, journal impact factors, third-party funding, or other widespread metrics of institutional performance. Many interviewees considered the included topics timely, as they related to ongoing discussions in the field regarding responsible research and contemporary science policy.

#### Clear presentation

Many interviewees indicated they appreciated the transparent overview. They liked the interactive features of the dashboard (e.g., switching between percentages and absolute numbers, hover-over fields with limitations) and appreciated the fact that it gave them a good overview whilst not overburdening the viewer with information.

### Weaknesses

#### Lack of an overall framework

Several participants indicated that they missed an overall narrative that would justify the choice of metrics included. In addition, it was unclear to some interviewees how the metrics related to each other, or to the quality of research. By presenting these metrics, it seemed as if these metrics were the standard metrics for institutional assessment, and various participants indicated that they did not consider that accurate as the metrics were not backed by an overall legal or regulatory framework. This made participants question who decides what good metrics for responsible research are. Without such a consented framework, participants feared that the dashboard would not be accepted as a viable complementary or alternative approach to evaluate research institutions.

#### Methods and conceptualization difficult to understand

Several interviewees expressed difficulty in interpreting the methods by which the metrics were compiled and the limitations inherent to these methods. This included the fact that the metrics may change over time, e.g., the numbers for Green Open Access might change once more publications are made available. Various participants stressed that the way particular metrics were operationalized was unclear. Finally, participants found it difficult to understand the metrics’ denominators, indicating it was unclear whether all metrics pertained to the same dataset or to different (parts of the) dataset(s), and whether the dashboard used all relevant available data sources (e.g., all eligible study registries), to provide a less biased measurement.

#### Possibly outdated

Some participants were concerned that metrics on robustness of animal research or Open Science stemming from publications in 2018 would present an outdated picture. They pointed to various developments in those areas in recent years and interviewees expected that publications from 2019 and 2021 would show higher robustness and Open Science scores. Although the fact that the data were taken from 2018 was a feature of the proof-of-principle dashboard, interviewees’ concerns underscore the need to continuously integrate new information into the dashboard, ideally in an automated manner.

### Opportunities

#### Initiating change

The dashboard was considered a good tool to start an evidence-based conversation on how to improve responsible research practices and about a more holistic evaluation of research institutions, i.e., assessing different institutional facets and not merely citations or impact factors. Some interviewees remarked that the dashboard could provide more information about how one could induce change, and aid in the development of concrete interventions. The dashboard would then, in their view, need to be presented to organizational leadership on a regular basis to fuel a discussion about efforts to improve. In addition, participants thought the dashboard could promote collective improvement efforts (or ‘healthy competition’) that would enable institutions to learn from each other.

#### Benchmarking over time

Several interviewees stressed that the dashboard should include the possibility to benchmark oneself over time. Whereas this was possible for some indicators, many interviewees believed that having this data over time for all metrics could help institutions to evaluate whether interventions to improve their performance on particular metrics were successful.

#### Internal usage only

When thinking about how to prevent possible detrimental consequences of benchmarking, many interviewees stressed that it would be most responsible if the dashboard would be used for internal purposes only. The dashboard’s information would not be accessible to anyone outside the institution, except if the institutional leadership chose to publicize some information. Some interviewees suggested that the dashboard should go public with a 2- or 3-year delay, which would give institutions the opportunity to improve first.

#### Tailoring the dashboard

Another opportunity that various interviewees raised was tailoring the dashboard to the institutional strategy. This would mean that different institutions would have different dashboards with different metrics, allowing institutions to create focus areas for a specific timeframe (e.g., in 3 years’ time, at least 60% of our publications should have open data). Related to this, some interviewees remarked that it would be helpful to link to more fine-grained levels of information, e.g., on a particular center within the research institution or a research field level. They remarked that they would appreciate the opportunity to correct or at least comment on the numbers (e.g., providing a reason why an institution performs badly on trial reporting), or to withdraw from the dashboard.

#### Complementing the dashboard with other indicators

Various interviewees discussed additional metrics they thought might be included in the dashboard. Two of the most often mentioned suggestions were the percentage of publications that were first posted as a preprint and conflict of interest statements in publications. Interviewees involved in clinical trials thought a metric on the overall number of clinical trials conducted could provide useful context. They also suggested metrics related to patient engagement in clinical research. In relation to animal research, some interviewees thought a metric for preregistration of animal studies could be useful, while others mentioned the choice of the model system as an important metric for the generalizability and translation of research. More broadly speaking, various interviewees mentioned metrics related to diversity, such as the percentage of female researchers or female principal investigators at a particular institution, or metrics related to the societal value of research, such as uptake of research by the media, policy documents, clinical guidelines, or patents.

#### Communicating metrics’ performance

Different interviewees remarked that they missed statistics on metrics’ performance. They would like to see uncertainty indicators such as confidence intervals, so that they could assess when an institute would perform better or worse than all institutions together. They also asked for numbers on the sensitivity and specificity of the sampling and classification approaches.

### Threats

#### Putting institutions in a bad light

Many interviewees feared that making the dashboard public in its current form would put institutions in a bad light. Whereas they thought some form of benchmarking could be helpful, they worried that the dashboard could be used as a tool to name and shame institutions, which could in turn have detrimental consequences for funding or for an institution’s reputation among the public. Some interviewees also mentioned that institutions should not be blamed for not meeting a metric’s requirement in the absence of sufficient infrastructure in place (e.g., a data sharing platform) that would allow institutions to comply. A few interviewees also pointed out that there is no inherent need for a comparison, since it is already self-evident that institutions should do their best to score high on these metrics.

#### Incorrect interpretation

Various interviewees were concerned that the metrics would be misinterpreted, whether intentionally or unintentionally. Especially under precious time, stakeholders might not appreciate the metrics’ limitations and instead take them at face value. This might result in inflating minor differences and risk the resource-heavy implementation of policies based on little understanding of the metrics. Additionally, some interviewees wondered whether other German UMCs were the right comparator, as there are large differences between the institutions.

#### Gaming metrics

Finally, various interviewees thought that the dashboard and the indicators therein could be gamed. Researchers at the respective institutions might feel that they must score high on these metrics and might get creative in the ways to score high, without living up to the ideals included in the respective metrics. More generally, some interviewees felt that boiling down these important issues to numeric indicators was not without risks.

## Discussion

We enquired stakeholders’ views on a dashboard that displayed the adoption of responsible research practices on a UMC-level. Overall, interviewees were positive about the dashboard approach, indicating that it allowed them to “talk reality”, i.e., seeing the actual data corresponding to their own UMC. Various interviewees missed a justification for why these specific metrics and no other potentially relevant metrics for responsible research were included. Some interviewees expressed difficulty in understanding how the metrics were derived from the data. Different interviewees believed the dashboard could be instrumental in sparking behavioral change on an institutional level and hoped the dashboard would include more diverse metrics in the future. The main fear among interviewees was that making the dashboard public would risk metrics being taken at face value and could put UMCs in a bad light.

### Interpretation

The SWOT matrix contains some inherent tensions. For example, opportunities ‘internal usage only’ and ‘initiating change’ (i.e., fueling competition among UMCs on who scores highest on certain metrics) represent two sides of the same issue in the contemporary research culture. On the one hand, Open Science is an increasingly popular topic and there may be the fear of being left behind. On the other hand, being an early adopter can be risky as it may show that you do not perform as well as you would like to believe. In addition, the data that the metrics are based on comes from public databases (e.g., clinicaltrials.gov or PubMed, see [26] for an overview of various metrics), and most of the tools used to create the metrics are publicly available as well. What then is the ideal level of openness or publicness of this information? We are presented with the dilemma that disclosing the dashboard to a select group of institutional leadership would be safer but would risk that nothing happens. Making the dashboard public would be more likely to spark discussion but could harm the reputation of some institutes. The sensitivity of the topic underscores the need to collaborate closely with the respective research performing organizations when implementing a dashboard for responsible research.

Interestingly, no stakeholder objected to the use of these alternative metrics (note that some were very critical about how they should be used and what sources they were based on). This reflection signals some level of acceptance of responsible research practice in the contemporary assessment culture. Some stakeholders commented explicitly on how these metrics reflected practices that could serve science as a whole. A lot of discussion was devoted to other metrics that could be added, especially on how science serves society, e.g., societal impact or patient-engagement. Part of the discussion was also devoted to inherent risks of quantification. In particular, some stakeholders were concerned that by making some practice measurable, one often ends up with a proxy for a particular practice that may itself be unrelated to the quality of the research.

We discerned some differences between different stakeholder groups. Whereas support staff and UMC leadership raised various practical concerns (e.g., these metrics can put UMCs in a bad light, it is hard to fully understand these metrics) experts in responsible research were more concerned about the lack of an overall framework to fit this dashboard. This may reflect a shift in community discussion; it is clear that research has to be assessed differently, the contemporary challenge is the design and implementation of more responsible assessment. It also raises the question of whether an overall framework is desirable, as it may overshadow relevant disciplinary differences within biomedical research.

### Contextualization

#### Overarching frameworks

There are a variety of frameworks available that are all applicable to the topic of responsible research practices. Broad examples include the TOP guidelines [27] that include, beyond data and code transparency, study preregistration and openness about materials, and focus on what journals incentivize. Another broad example, this time focused on individual researchers, are the Hong Kong Principles [7], where explicit attention is paid to peer review and, more recently, diversity [28]. More specific frameworks include ARRIVE for animal research [29], or the Cochrane Risk of Bias tool for clinical trials [30].

Some of our metrics find their origin in regulatory frameworks, such as the mandated registration of drug trials in EudraCT [31]. In addition, the World Health Organization has specified that results of clinical trials should be published in a relevant journal within two years of trial completion [32, 33]. The Declaration of Helsinki [34] calls for all research involving human subjects to be prospectively registered and published.

That said, no framework for responsible research seems broad enough or has received universal or consented support. It is questionable whether such a framework will be developed, and it raises the question of who should oversee its development. A potential starting point would be the UNESCO recommendations of Open Science [35]. An alternative approach, on a more local level, could be to involve a group of UMCs in a consensus-building procedure to find a set of indicators for responsible research that these particular UMCs agree upon (see [36]).

#### Dashboards for institutions

We witnessed a surge in dashboards for responsible research and related topics in recent years. Some have a broad focus, such as the European Commission Open Science Monitor, project TARA within the context of DORA or the French Open Science Monitor that display broad disciplinary fields [37–39]. Others focused on (and ranked) individual researchers in terms of transparency, such as the Curate Science Transparency leaderboard [40]. Some of our interviewees questioned whether an entire UMC is the right level for developing such a dashboard. It is interesting in this context that the DFG’s revision of the Guidelines for Safeguarding Good Research Practice [41] includes explicit chapters on responsibilities for heads of research organizations. We believe that a dashboard approach could support heads of research organizations to empirically monitor the implementation of the revised code of conduct.

### Strengths and weaknesses of the study

This is the first comprehensive map of stakeholders’ views on a dashboard that visualizes UMC performance using metrics for responsible research. We interviewed both stakeholders within the German biomedical research landscape that are familiar with existing UMC incentives, as well as those that worked at universities outside Germany who could provide an international perspective.

There are some limitations to acknowledge. First, the stimulus we used regarded a proof-of-principle dashboard and therefore some SWOTs may be specific to our dashboard and less generalizable. We tried to mitigate this by using interviewees’ feedback on our dashboard to highlight broader issues, such as the lack of an overall consented framework governing responsible research on an institutional level.

Second, stakeholders who are generally skeptical of the idea to evaluate UMCs differently might decline the invitation to participate in a study like ours. Although some stakeholders were very critical, the views presented in this paper might be rosier than the average stakeholder. We tried to invite stakeholders from outside our network, especially German UMC leadership (*n* = 15), but despite various reminders, they were not willing to participate or did not reply.

Third, we invited primarily academic stakeholders. The views presented here thus reflect primarily those that could be useful for UMCs or other academic institutions. Conceptualized more broadly, stakeholders would include more external parties such as government regulators, science journalists, members of the public, and explicit critics of academic reform. Some of these views could be even more positive than those presented in the current paper, others more negative, yet their absence should be noted.

## Conclusions

We described what stakeholders considered the strengths, weaknesses, opportunities, and threats of a dashboard displaying the adoption of responsible research practices on an institutional level. Our aim was descriptive and exploratory; readers should be aware there is no hierarchy of importance among the different subtopics and their inherent tensions should be weighed with caution. Overall, stakeholders appreciated the focus on behaviors that allowed them to see where a UMC stands but pointed to the lack of a justification for the metrics included. They feared institutions might be put in a bad light, underscoring the need for close collaboration with research institutions when implementing alternative approaches to evaluate research institutions.

## Supporting information

S1 File Communication to interview participants. Invitational e-mail, information letter, informed consent, further correspondence).
(DOCX)

S2 File Proof-of-principle dashboard in screenshots with metric descriptions. See also tutorial: https://www.youtube.com/watch?v=VDdljq5zI9E.
(DOCX)

S3 File Topic guide. Please note: *Italics* are alternative phrasings.
(DOCX)

S4 File Code tree. *Note*: This code system consists of three levels. The SWOTs in bold (e.g., “Strengths”, highest level), the themes in *italics* (e.g., “data and behavior driven” medium level–different topics that could be interpreted as SWOTs), and subcodes in regular font (e.g., “pick a core set of metrics”, lowest level–subcomponents of a theme).
(DOCX)
