# Supplementary material for: Stakeholders’ views on an institutional dashboard with metrics for responsible research
Source: PLoS One. 2022 Jun 24;17(6):e0269492. doi: 10.1371/journal.pone.0269492 (PMC9231768; doi:10.1371/journal.pone.0269492)
Supplement: S1 File — Invitational e-mail, information letter, informed consent, further correspondence). (DOCX) [file pone.0269492.s001.docx]

**Supporting information 1.** Communication to interview participants (i.e., invitational e-mail, information letter, informed consent, further correspondence)

Dear …,

We would like to invite you to participate in an interview to gain your expert view on the strengths and weaknesses of a new type of **dashboard** that displays the adoption of **measures for robust and open science** at a specific University Medical Center (UMC).

We developed such a proof-of-principle dashboard in the BMBF-funded project BRAVO. The dashboard displays UMC-specific information on a) Open Science, b) the registration and reporting of clinical trials, and c) the reporting of robustness measures in animal research. We are now seeking feedback on this dashboard from various stakeholders such as funders, scientists, library staff, and UMC leadership.

TAILOIRED TO FUNDERS:
Given your role as a funder in incentivising responsible research practices, we would very much appreciate your feedback on this dashboard.

TAILOIRED TO NOVEL METRICS EXPERTS:
As your work on [paper] has really shaped the debate on research assessment, we would very much appreciate your feedback on this dashboard.

TAILOIRED TO LIBRARY STAFF:
In your role at the library and expertise in assessing how your institute is performing research-wise, we would very much appreciate your feedback on this dashboard.

TAILOIRED TO UMC LEADERSHIP:
In your role as [dean/department lead/etc.], we would very much appreciate your feedback on this dashboard, as it was created to help German university medical centres visualise the adoption of responsible research practices at their institution.

By participating in this interview study, your views may help inform future policy making on research assessment. The interview will focus on the following core questions:

- What would you consider are the strengths and weaknesses of this dashboard approach?
- Which metric(s) would you find most informative to support decision making, and why?

Could you please let us know by replying to this email if you would be willing and available to participate in an (online) interview? We will then schedule an interview time that fits your calendar.

If you are willing to participate, you will be sent a link to the proof-of-principle dashboard as well as a short explanatory tutorial. The interviews will be conducted online between March and May and last between 30-45'. We offer an allowance of €150 for your time and effort. Please see the attached information letter for more background information on the study, a link to the study protocol, as well as detailed information on confidentiality and data protection.

If you know any other suitable colleagues who would be interested, please share their name with us. Should you have any questions, please don't hesitate to contact me, Tamarinde Haven, via [tamarinde.haven@charite.de](mailto:tamarinde.haven@charite.de).

Thank you very much for your consideration, on behalf of Prof. Daniel Strech and the BRAVO study team at the BIH QUEST Center,

Tamarinde Haven

Sehr geehrte/r Herr/Frau...,

wir möchten Sie gerne zur Teilnahme an einem wissenschaftlichen Interview einladen, um Ihre Expertenmeinung zu den Stärken und Schwächen eines neuartigen **Dashboards** zu erfahren, das die Umsetzung von **Maßnahmen für robuste und offene Wissenschaft** an Universitätsklinika (University Medical Centers, UMC) darstellt.

Wir haben dieses Proof-of-Principle-Dashboard in dem BMBF-geförderten BRAVO-Projekt entwickelt. Das Dashboard zeigt UMC-spezifische Informationen zu a) Open Science, b) der Registrierung und Veröffentlichung klinischer Studien und c) Informationen zur Robustheit in Veröffentlichungen der Tierforschung. Wir sind nun am Feedback verschiedener Stakeholder zu diesem Dashboard interessiert, zum Beispiel Geldgebern, Wissenschaftlern, Bibliotheksmitarbeitern und dem Leitungspersonal aus der Universitätsmedizin.

VERSION FÜR GELDGEBER:

Angesichts der Rolle, die Sie als Geldgeber für die Förderung verantwortungsvoller Forschungspraktiken spielen, würden wir uns sehr über Ihr Feedback zu diesem Dashboard freuen.

VERSION FÜR EXPERTEN DES THEMAS „NEUE METRIKEN“:

Da Ihre Arbeit an den [paper] die Debatte über die Bewertung von Forschungsleistungen maßgeblich geprägt hat, würden wir uns sehr über Ihr Feedback zu diesem Dashboard freuen.

VERSION FÜR BIBLIOTHEKSMITARBEITER:

In Ihrer Rolle in der Universitätsbibliothek, und mit Ihrer Expertise in der Bewertung der Forschungsleistungen Ihrer Institution, würden wir uns sehr über Ihr Feedback zu diesem Dashboard freuen.

VERSION FÜR LEITUNGSPERSONAL DER UNIVERSITÄTSMEDIZIN:

In Ihrer Rolle als [Dekan/Institutsdirektor/Klinikdirektor/etc.] würden wir Ihr Feedback zu diesem Dashboard sehr schätzen, da es erstellt wurde, um deutschen Universitätsklinika dabei zu helfen, die Einführung verantwortungsvoller Forschungspraktiken an ihrer Institution zu visualisieren.

Indem Sie an dieser Interviewstudie teilnehmen, können Ihre Ansichten dazu beitragen, zukünftige politische Entscheidungen zur Bewertung von Forschungsleistungen zu beeinflussen. Das Interview wird sich auf die folgenden Kernfragen konzentrieren:

- Was sind Ihrer Meinung nach die Stärken und Schwächen des Dashboard-Ansatzes?
- Welche Metrik(en) fänden Sie am informativsten, um die Entscheidungsfindung zu unterstützen, und warum?

Bitte teilen Sie uns mit, ob Sie bereit und verfügbar wären, an einem (Online-)Interview teilzunehmen, indem Sie auf diese E-Mail antworten. Wir werden dann einen Interviewtermin vereinbaren, der Ihnen passt.

Wenn Sie bereit sind, an dem Interview teilzunehmen, erhalten Sie einen Link zum Proof-of-Principle-Dashboard sowie ein kurzes erklärendes Tutorial. Die Interviews werden zwischen März und Mai online durchgeführt und dauern 30–45 min. Wir bieten eine Aufwandsentschädigung in Höhe von 150€ an. Weitere Hintergrundinformationen zur Studie, einen Link zum Studienprotokoll sowie detaillierte Informationen zu Vertraulichkeit und Datenschutz entnehmen Sie bitte dem beigefügten Informationsschreiben.

Falls Sie weitere geeignete Kollegen kennen, die Interesse hätten, teilen Sie uns bitte deren Namen mit. Sollten Sie weitere Fragen haben, zögern Sie bitte nicht, mich, Tamarinde Haven, über tamarinde.haven@charite.de zu kontaktieren.

Im Namen von Prof. Dr. Dr. Daniel Strech und dem BRAVO-Studienteam des BIH QUEST Center bedanke ich mich herzlich für Ihre Hilfe,

Tamarinde Haven

**DASHBOARD VISUALISING METRICS FOR ROBUST AND OPEN RESEARCH**
***Study information and consent form***

Current indicators for scientific performance are citation metrics and secured third party funding. In order to increase value and reduce waste in the biomedical sciences^[[1]](#footnote-1)^, it has been argued that research institutions should move away from traditional metrics of research evaluation and towards metrics that reflect responsible research practices^[[2]](#footnote-2)^, such as the timely reporting of clinical trial results and open access publications^[[3]](#footnote-3)^.

The BMBF funded project ‘BRAVO’ developed a dashboard (in a proof-of-principle version) that allows UMCs to visualize the adoption of responsible research practices at their institution by means of ‘metrics’ that relate to open science, the registration and reporting of clinical trials, and the robustness of animal studies. This proof-of-principle version serves to illustrate the main features of the dashboard, which is still in development.

In order to understand how stakeholders view the Strengths, Weaknesses, Opportunities, and Threats (SWOTs) of this dashboard approach, we want to conduct in-depth interviews with these main aims:

1. To determine and better understand stakeholders’ views on this proof-of-principle dashboard displaying metrics (that visualize)/as indicators of responsible research practices

2. To better understand participants’ views on these different metrics intended to display the adoption of responsible research practices on a research-institute level

**How your input will be used**

The interviews will be recorded with a recording device or software and will be transcribed by members of the study team of external employees of a transcription service. External personnel will not receive any information about participants’ identity or institution; the audio file will be destroyed after transcription, in accordance with data protection laws. The results will be used in a scientific paper, where we will illustrate our findings with quotes from the interview. These quotes are anonymized, but you might be able to recognize your own citations. The goal of the study would be an overview of the strengths, weaknesses, opportunities and threats associated with this dashboard approach and its associated metrics. You can find our full study protocol here: <https://osf.io/ny8az/>.

Prof. Dr. Dr. Daniel Strech

Deputy Director QUEST Center

Tel 030 450 543 068

Fax: 030 450 7543999

daniel.strech@bihealth.de

28. Sep. 2021

**Voluntariness**

Participation in the interviews is voluntary. You have the option at any time to cancel an interview and withdraw your consent to a recording and transcription of the interview without incurring any disadvantages.

**Data Protection Note**

By signing the consent form, you agree for members of the study team to collect and process your personal data, for the purposes of the aforementioned study. The principal investigator, Prof. Dr. Dr. Daniel Strech, is the responsible party for data processing according to the EU General Data Protection Regulation (GDPR).

The pseudonymized research data as well as the identifying data will be separately stored and processed on Charité drives, conforming to the GDPR and the Berlin Data Protection Law. Only members of the study team will have access to these data. After the retention period of 10 years (according to good scientific practice guidelines) has ended, we will delete or anonymize the identifying data. Please not that from then on, no withdrawal of consent, no information, change or deletion is possible, since we cannot connect you to the data.

You have the right to receive information (including a free copy) about all of your personal data from the principal investigator. You also have the right to withdraw your consent to the data processing at any time; in the case of withdrawal, you can also ask for deletion of your personal data. Please note that the legality of the data processing that has happened until then is left untouched: i.e., those data that have already been used for scientific publications are not subject to the withdrawal. To exercise these rights, please contact the principal investigator under the contact details below.

**Responsible Principal Investigator:** Prof. Dr. Dr. Daniel Strech, QUEST Center for Transforming Biomedical Research, Berlin Institute of Health, Translational Research Unit of the Charité – Universitätsmedizin Berlin, Charitéplatz 1, 10117 Berlin, Germany, phone: +49 30 450 543 068, email: daniel.strech@bihealth.de, website: [www.charite.de](http://www.charite.de)

**Data Protection Officer:** You can always direct questions about the storage and processing of your data, or about your rights for data protection, to the Data Protection Officer of the Charité: Charité – Universitätsmedizin Berlin, Data Protection Office, Frau Janet Fahron, Charitéplatz 1, 10117 Berlin, phone: +49 30 450 580 016, email: [datenschutz@charite.de](mailto:datenschutz@charite.de)

**Berlin Representative for Data Protection and Freedom of Information**: You have the right to complainat the Berlin Representative for Data Protection and Freedom of Information, which is the supervisory body for data protection. You have the right to veto at the responsible authority if you are under the impression that your data are being processed illegally: Berlin Representative for Data Protection and Freedom of Information (Berliner Beauftragte für Datenschutz und Informationsfreiheit), Friedrichstr. 219, 10969 Berlin, phone: +49 30 13889-0, email: [mailbox@datenschutz-berlin.de](mailto:mailbox@datenschutz-berlin.de)

**Declaration of consent**

I hereby consent to participate in a semi-structured interview as part of the BRAVO project. I understand that my personal data will be collected, recorded, stored and processed for the purpose of the aforementioned study in a pseudonymized way. I was explained the type and aims of the study. I understand that the results of the study are published in an anonymized form, which does not allow for my direct identification. I had ample opportunity to ask the study team questions. I am aware that my participation is voluntary and that I, at any time, have the right to withdraw my consent without providing any reasons and without any disadvantages for myself, and that I can withdraw my consent to further processing of my data, as well as ask for their destruction.

__________________ ____________ ____________________________________________

NAME DATE SIGNATURE

Link to dashboard + tutorial

Dear ________,

Thank you for your willingness to participate in the BRAVO interview study. Would any of the following days/times suit your calendar:

DATES/TIMES 
 
The interview will take place via Microsoft Teams. We will send you a reminder 1 day before the interview.

Please find the dashboard here (if the hyperlink does not work, copy-paste the following URL into your browser: [LINK]). You might want to watch this brief [tutorial](https://youtu.be/VDdljq5zI9E) where we explain how to navigate the dashboard. The dashboard is still under development, please do not share this link with others.

We look forward to talking with you on.

Kind regards,

Tamarinde Haven

Link to dashboard + tutorial (**German**)

Sehr geehrte/r ________,

vielen Dank für Ihre Bereitschaft, an der BRAVO-Interviewstudie teilzunehmen! Bitte teilen Sie uns mit, ob Sie einen – und falls ja, welchen – der angegebenen Termine Sie wahrnehmen könnten:

DATEN/ZEITEN 
 
Wir würden das Interview gerne auf Englisch durchführen, bitte teilen Sie uns daher kurz mit, ob das in Ordnung für Sie ist. Der Termin findet via Microsoft Teams statt. Wir schicken Ihnen einen Tag vor dem Interview eine Erinnerungs-E-Mail.

Unser Dashboard finden Sie bitte hier (falls der Link nicht funktionieren sollte, kopieren Sie bitte die folgende URL und fügen sie in der Adresszeile Ihres Browsers ein: [LINK]). Wir empfehlen Ihnen, [dieses Tutorial](https://youtu.be/VDdljq5zI9E) (in englischer Sprache) zu schauen, in den wir erklären, wie Sie das Dashboard benutzen. Das Dashboard befindet sich derzeit ncoh in Entwicklung, daher bitten wir Sie, den Link nicht mit anderen zu teilen.

Wir freuen uns auf das Gespräch mit Ihnen!

Mit herzlichen Grüßen,

Tamarinde Haven

1. Macleod, M. R., Michie, S., Roberts, I., Dirnagl, U., Chalmers, I., Ioannidis, J. P. A., Salman, R. A.-S., Chan, A.-W., & Glasziou, P. (2014). Biomedical research: Increasing value, reducing waste. *The Lancet*, *383*(9912), 101–104. <https://doi.org/10.1016/S0140-6736(13)62329-6> [↑](#footnote-ref-1)
2. Ioannidis JPA (2014) How to Make More Published Research True. PLOS Medicine 11(10): e1001747. <https://doi.org/10.1371/journal.pmed.1001747>

   Moher, D., Bouter, L., Kleinert, S., Glasziou, P., Sham, M. H., Barbour, V., Coriat, A.-M., Foeger, N., & Dirnagl, U. (2020). The Hong Kong Principles for assessing researchers: Fostering research integrity. *PLOS Biology*, *18*(7), e3000737. <https://doi.org/10.1371/journal.pbio.3000737> [↑](#footnote-ref-2)
3. Begley, C. G., Buchan, A. M., & Dirnagl, U. (2015). Robust research: Institutions must do their part for reproducibility. *Nature*, *525*(7567), 25–27. <https://doi.org/10.1038/525025a>

   Flier, J. (2017). Faculty promotion must assess reproducibility. *Nature*, *549*(7671), 133–133. <https://doi.org/10.1038/549133a>

   Strech, D., Weissgerber, T., & Dirnagl, U. (2020). Improving the trustworthiness, usefulness, and ethics of biomedical research through an innovative and comprehensive institutional initiative. *PLOS Biology*, *18*(2), e3000576. https://doi.org/10.1371/journal.pbio.3000576 [↑](#footnote-ref-3)
