# Supplementary material for: Stakeholders’ views on an institutional dashboard with metrics for responsible research
Source: PLoS One. 2022 Jun 24;17(6):e0269492. doi: 10.1371/journal.pone.0269492 (PMC9231768; doi:10.1371/journal.pone.0269492)

**Supporting information 2.** Proof-of-principle dashboard (in screenshots)

Screenshot 1. Landing page with explanation


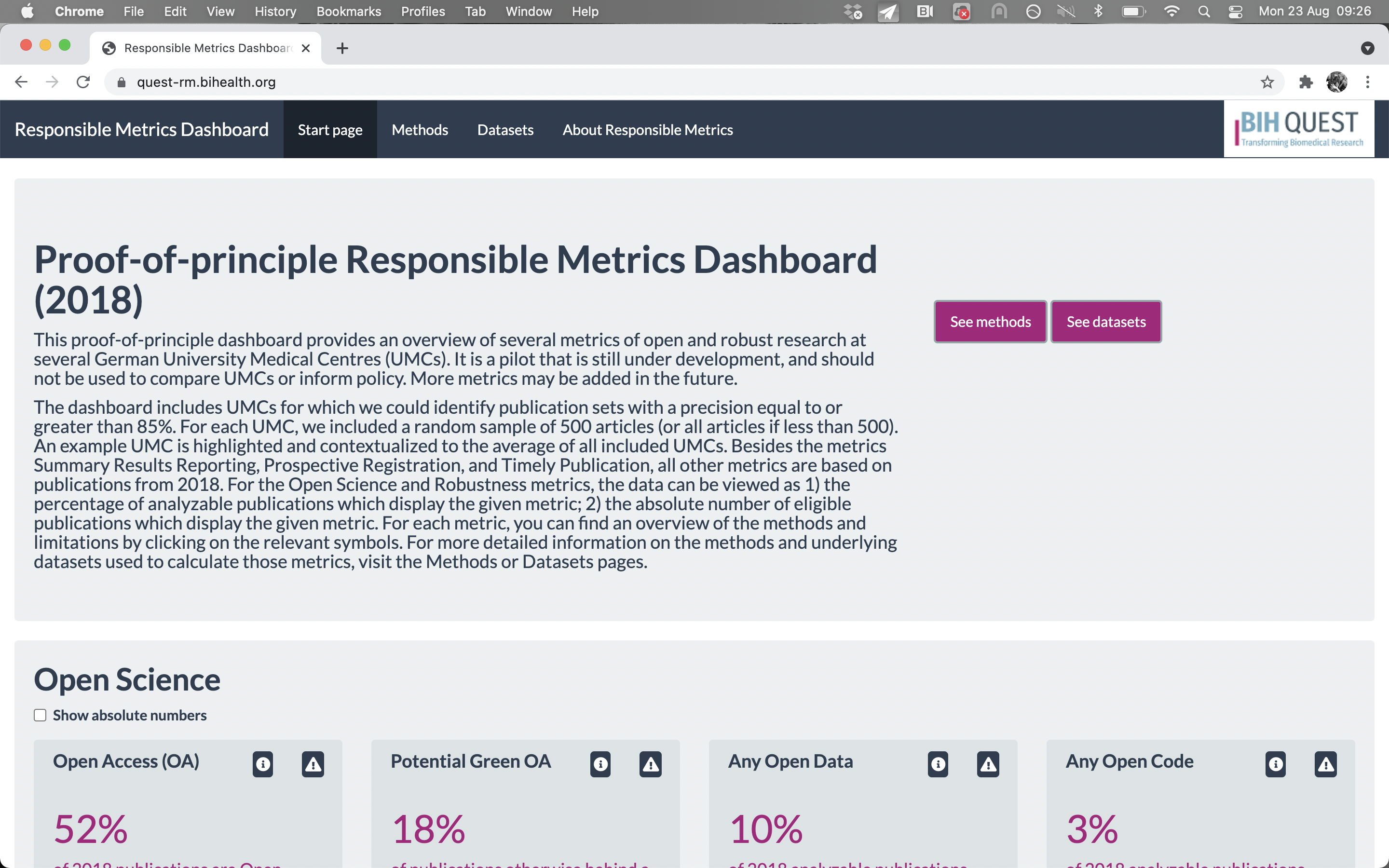


**PUBLICATION SEARCH**

Many of the assessed metrics are publication-based metrics. To assess those metrics on the institutional level, we first had to identify publications that can be assigned to UMCs in Germany. We searched Web of Science for publications published in 2018 with at least one author at each UMC. We used the organisation-enhanced index of the Web of Science Core Collection to disambiguate author affiliations. As a proxy for the publication output of UMCs, we identified biomedical publications at the aforementioned institutions using a combination of biomedical journal-level subject categories in Web of Science and article-level categories in Dimensions. The results were filtered for the following document types: 'Article' and 'Review'. We included publications in all languages. Web of Science searches and extractions were performed between 14/08/2020 and 22/09/2020. The Dimensions query was performed on 22/09/2020.

To evaluate the precision of our approach, we performed a manual check of a random sample of publications per UMC. A [detailed protocol](https://osf.io/a248e/) of our precision checks is openly available in OSF. Briefly, 50 publications per UMC were manually checked as to whether any author is affiliated to the medical faculty of the university of question. Based on these results, we generated a proof-of-principle dataset which includes UMCs for which we could identify publication sets with a precision equal to or greater than 85% (n=13 UMCs). For each UMC, we included a random sample of 500 articles (or all articles if less than 500). Reviews were excluded from this proof-of-principle dataset as most metrics are based on articles. An example UMC is highlighted and contextualized to the average of all UMCs included in the proof-of-principle dataset.

Screenshot 2. Metrics related to Open Science


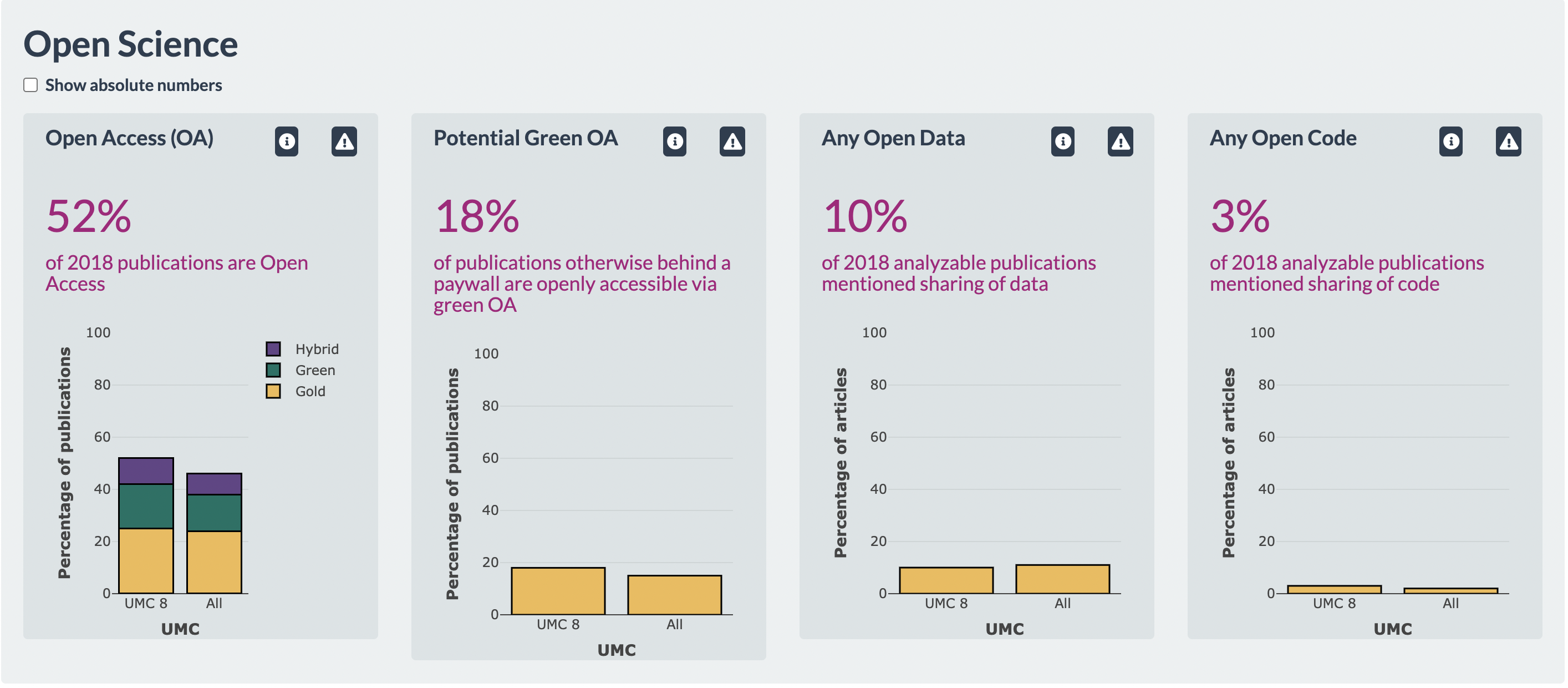


**Open Access**

***What it measures:***

A lot of valuable research, much of which is publicly funded, is hidden behind paywalls. Open Access (OA) makes research articles available online, free of charge and most copyright barriers. The free, public availability of research articles accelerates and broadens the dissemination of research discoveries. OA also enables greater visibility of research and makes it easier to build on existing knowledge. Research funders are increasingly encouraging OA to maximise the value and impact of research discoveries.

***How it was calculated:***

Using the obtained list of institutional publications, we queried the Unpaywall database via its [API](https://unpaywall.org/products/api) to obtain information on the OA status of the publications. Unpaywall is today the most comprehensive database of OA information on research articles. It can be queried using publication DOIs. Publications can have different OA statuses which are color-coded. Gold OA denotes a publication in an OA journal. Green OA denotes a freely available repository version. Hybrid OA denotes an OA publication in a journal which offers both a subscription based model as well as an OA option. Bronze OA denotes a publication which is freely available on the journal page, but without a clear open license. These can be articles in a non-OA journal which have been made available voluntarily by the journal, but which might at some stage lose their OA status again. Thus, we only consider the OA categories gold, green and hybrid for this dashboard. As one publication can have several OA versions (e.g. a gold version in an OA journal as well as a green version in a repository), we define a hierarchy for the OA categories and for each publication only assign the OA category with the highest priority. We use a hierarchy of gold - hybrid - green (journal version before repository version), as also implemented in the Unpaywall database itself. After querying the Unpaywall API for all publication DOIs, we group the results by OA status.

One important point that has to be considered with OA data is that the OA percentage is not a fixed number, but changes over time. This is due to the fact that repository versions are often made available with a delay, such that the OA percentage for a given year typically rises retrospectively. Thus, the point in time at which the OA status is retrieved is important for the OA percentage. The current OA data was retrieved using (with [UnpaywallR](https://github.com/NicoRiedel/unpaywallR)) on: 28/02/2021.

***Limitations:***

Unpaywall only stores information for publications which have a DOI assigned by Crossref. Articles without a Crossref DOI have to be excluded from the OA analysis.

**Potential Green Open Access**

***What it measures:***

This metric measures how many paywalled publications with the potential for green OA have been made openly accessible in a repository. In many cases, journal or publisher self-archiving policies allow researchers to make the accepted version of their publication openly accessible in a repository after an embargo period.

***How it was calculated:***

We queried the Unpaywall API (with [UnpaywallR](https://github.com/NicoRiedel/unpaywallR)) and applied the following hierarchy to identify publications only accessible via green OA: gold - hybrid - bronze - green. To identify paywalled publications with the potential for green OA, we filtered our dataset for paywalled publications and queried the [Shareyourpaper.org permissions API](https://shareyourpaper.org/permissions/about#api) (Open Access Button) to obtain article-level self-archiving permissions based on journal or publisher policies. Publications were considered to have the potential for green OA if an authoritative permission was found for archiving the accepted version in an institutional repository and the embargo (if any) had elapsed by the query date.

***Limitations:***

We only included publications which have an authoritative permission in the Shareyourpaper.org database. The date at which a publication can be made openly accessible via self-archiving depends on the publication date and the length of the embargo (if any). Therefore, the number of potential green OA research articles will change over time. The Shareyourpaper.org permissions API was queried on 28/02/2021. The Unpaywall database was queried with the aforementioned OA hierarchy on 11/03/2021.

**Open Data and Open Code**

***What it measures:***

The Open Data and Open Code metrics measure how many publications share their raw research data or analysis code along with the publication. Openly shared data and code makes research more transparent, as research findings can be reproduced. Additionally, shared datasets can be reused and combined by other scientists to answer new research questions. The definition of Open Data used here is a low barrier definition. Only a part of the raw data underlying a study has to be freely available and no further quality criteria such as the FAIR criteria are checked. Note also that data sharing is not possible for all studies, for example if there is no dataset to be shared or if the data cannot be shared, e.g. due to privacy concerns for patient data. Data sharing under restrictions is currently not considered, but this is planned in the future.

***How it was calculated:***

To identify publications which share research data or analysis code, we use the text-mining algorithm ODDPub (Code: <https://github.com/quest-bih/oddpub>, publication: <https://datascience.codata.org/article/10.5334/dsj-2020-042/>) developed by QUEST. ODDPub searches the publication full-text for statements indicating sharing of raw data or analysis code. It does however not check the shared data itself. A text-mining approach is necessary, as a standardized way of sharing and reporting Open Data does not yet exist, and no database offers sufficiently comprehensive information on shared datasets or code. To assess data and code sharing for UMC publications, we first downloaded the full-texts of the publications that were accessible to us using the Unpaywall and Crossref APIs. We screened those full-texts with ODDPub and calculated the percentages of Open Data & Code relative to the publications in English and available as full text.

***Limitations:***

Several limitations apply: Only full texts for Open Access publications or publications in journals to which we had a subscription could be retrieved (~78% of all detected publications). ODDPub only finds ~75% of all Open Data publications and finds false positive cases (no manual check of the results is done). ODDPub also does not verify that the indicated dataset is indeed available and whether the dataset fulfills our definition of Open Data. Open Data is not relevant for all publications, so we would not expect 100% of the publications to contain Open Data, not even in an ideal case. We considered all publications which had at least one author affiliated to one of the included UMCs. Authors with different project contributions and roles may have varing influence on the decision whether to make data or code available alongside a publication.

Screenshot 3. Metrics related to clinical trials


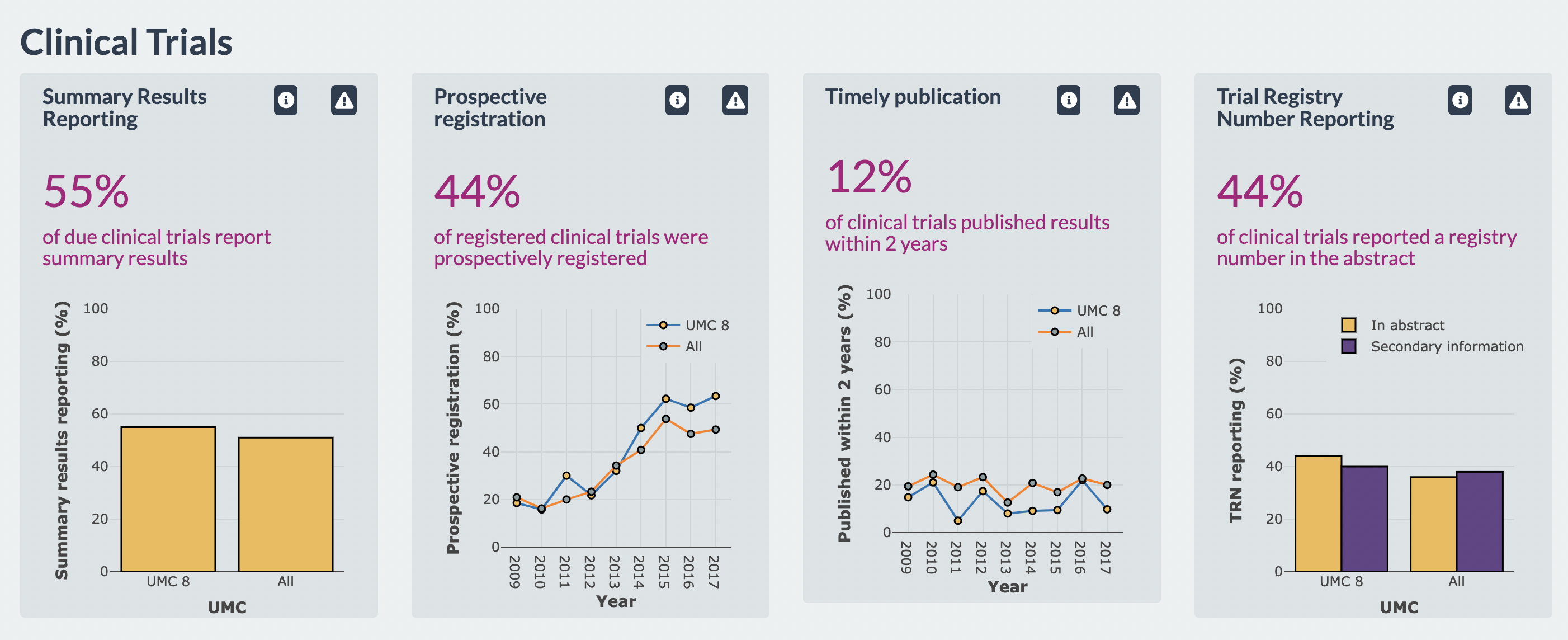


**CLINICAL TRIALS**

**Summary results reporting**

***What it measures:***

This metric measures how many clinical trials registered in the EU Clinical Trials Register that are due to report their results have already done so. A trial is due to report its results 12 month after trial completion. Clinical trials are expensive and have often many contributing patients. A fast dissemination of the trial results is crucial to make the evidence gained in those trials available. The World Health organization recommends publishing clinical trial results within one year after the end of a study.

***How it was calculated:***

The data were retrieved for all UMCs included in this proof-of-principle dataset from the [EU Trials Tracker](https://eu.trialstracker.net/) by the EBM DataLab.

***Limitations:***

While the EU Clinical Trials Register is one of the most predominant European trial registries, it is not the only available registry. There are other registries such as ClinicalTrials.gov. or the German Clinical Trials Registry, which are not considered here. Additionally, the EU Trials Tracker does not measure for how long the trials have been due. Finally, we only considered the latest data available in the EU Trials Tracker. We plan to include historic data in the future.

**Prospective registration**

***What it measures:***

This metric measures if the clinical trials are registered before the start date of the study, according to the information given on ClinicalTrials.gov or DRKS.de. The idea of prospective registration of studies is to make the trial specifications, including primary and secondary outcomes, publicly available before study start. Prospective registration adds transparency, helps protect against outcome switching.

***How it was calculated:***

We used the same methods as for the timely reporting metric to identify trials from UMCs. To assess if a study has been prospectively registered, we compare the date the study was first submitted to the registry with the start date given in the registry. As some of the earlier dates in the database only stated the month but not the exact day and to account for other possible delays we chose a conservative estimate of prospective registration and allow for a delay between start and registration date of up to 60 days.

***Limitations:***

For this metric, while there are other available registries, we only focused on ClinicalTrials.gov. Also, we rely on the information on ClinicalTrials.gov being accurate.

**Timely publication of results**

***What it measures:***

This metric measures how many clinical trials registered on ClinicalTrials.gov or DRKS.de reported their results either as a journal publication or as summary results on the trials registry within 2 years after completion. Trials completed between 2009 and 2017 were considered. A fast dissemination of the trial results is crucial to make the evidence gained in those trials available. The World Health organization recommends publishing clinical trial results within one year after the end of a study.

***How it was calculated:***

ClinicalTrials.gov and DRKS.de were searched for studies with one of the UMCs as the responsible party/sponsor or with a principal investigator from one of the UMCs. A manual search for published results was done, searching the registry, PubMed and Google. When calculating the time to publication, we only considered trials where we could track the full timeframe since completion. A part of these data were were previously published in the [IntoValue study](https://s-quest.bihealth.org/intovalue/). Detailed methods can be found in the [associated publication](https://www.sciencedirect.com/science/article/abs/pii/S0895435618310631?via%3Dihub).

***Limitations:***

Some detected publications might be missed in the manual search procedure as we only searched a limited number of scientific databases and did not contact the responsible parties. Furthermore, we did not include observational clinical studies in our sample. Additionally, we might overestimate the time to publication for some studies as we stopped the manual search after the first detected publication.

**Reporting of Trial Registration Number (TRN)**

***What it measures:***

Reporting of clinical trial registration numbers in related publications facilitates transparent linkage between registration and publication and enhances the value of the individual parts towards more responsible biomedical research and evidence-based medicine. The [Consolidated Standards of Reporting Trials (CONSORT)](https://www.sciencedirect.com/science/article/pii/S0140673607618352?via%3Dihub) as well as the [ICMJE Recommendations for the Conduct, Reporting, Editing, and Publication of Scholarly Work in Medical Journals](http://www.icmje.org/recommendations/) call for reporting 'trial registration number and name of the trial register'in both the full-text and abstract.

***How it was calculated:***

We developed an [open source R package](https://github.com/maia-sh/ctregistries) for the detection and classification of clinical trial registration numbers. Our regular-expression-based algorithm searches text strings for matches to TRN patterns for all PubMed-indexed and ICTRP-network registries. In a first step, we filtered the publication dataset for PubMed-classified human clinical trials. Then, we used the aforementioned package to detect and classify trial registration numbers in the PubMed secondary identifier metadata and abstract.

***Limitations:***

We identified human clinical trials based on the following search term in PubMed: 'clinical trial'[pt] NOT (animals [mh] NOT humans [mh]). However, we have not tested (1) the sensitivity of this PubMed search term (i.e., what proportion of true clinical trial publications are detected?); (2) the precision of this search term (i.e, what proportion of detected publications are not true clinical trials publications?). Furthermore, our algorithm does not distinguish true TRNs that do not resolve to a registration. Finally, the algorithm does not determine whether the TRN is reported as a registration for the publication's study (i.e., clinical trial result) or is otherwise mentioned (i.e., in a review, reference to other clinical trials, etc.)

Screenshot 4. Metrics related to animal research


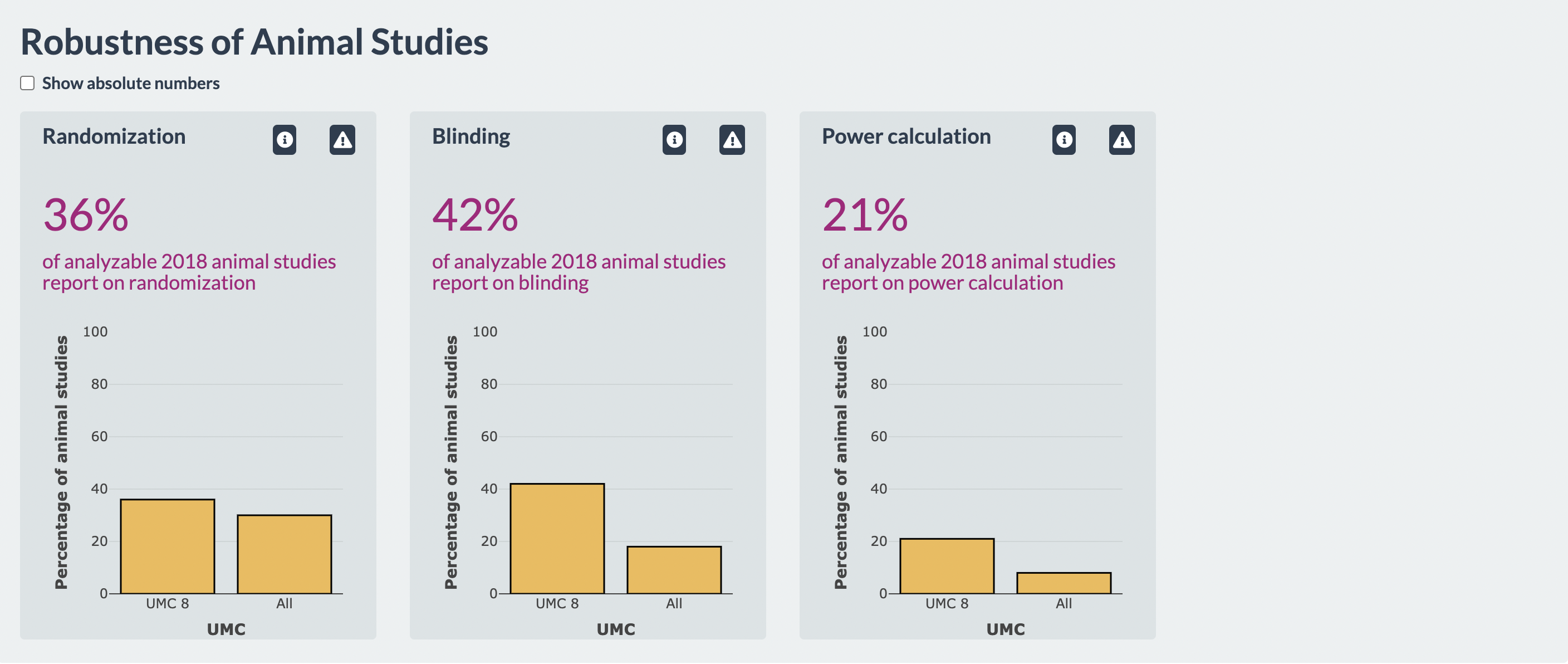


**ROBUSTNESS**

**Robustness of animal studies**

***What it measures:***

The robustness measures depend on the performance of the SciScore classifier (see [Menke et al. 2020](https://www.sciencedirect.com/science/article/pii/S2589004220308907) for more information on classifier performance analysis). Specifically, we focus on the following research parameters: reporting of investigator blinding, randomization of subjects, and sample size calculation.

***How it was calculated:***

In a first step, we filtered the publication dataset for animal studies based on a previously published [PubMed search query](https://www.ncbi.nlm.nih.gov/pmc/articles/PMC3104815/). The robustness measures were evaluated with [SciScore](https://www.sciencedirect.com/science/article/pii/S2589004220308907), an automated tool which evaluates research articles based on their adherence to rigour and reproducibility criteria.

***Limitations:***

There are several limitations: (1) Identification of animal studies. We identified animal studies based on a previously published PubMed search filter which has been shown to retrieve more records than the regular search method in PubMed (Limit: Animals). However, we did not test its sensitivity and precision in the context of this proof-of-principle dataset. Moreover, this PubMed search filter does not distinguish between publications in which animals are mentioned, and publications in which animals are the main research subject. It also does not identify animal studies in our publication set not indexed in PubMed. (2) Evaluation of robustness measures. For the purposes of this dashboard, we obtained SciScore data for publications in the PubMed Central (PMC) corpus. At this stage, we do not have this data for publications in our dataset not indexed in PMC. We also only included animal studies in English in the analysis. The robustness measures depend on the performance of the SciScore classifier (see more information on classifier performance analysis in [this publication](https://www.sciencedirect.com/science/article/pii/S2589004220308907?via%3Dihub#mmc1)). Finally, it is important to note that randomization, blinding, and sample size calculation may not always apply, especially in early-stage exploratory research (hypothesis-generating experiments). At present, we do not have a way of distinguishing these studies from confirmatory, hypothesis-testing experiments.

Screenshot 5. Demonstration “info icon” and “warning icon


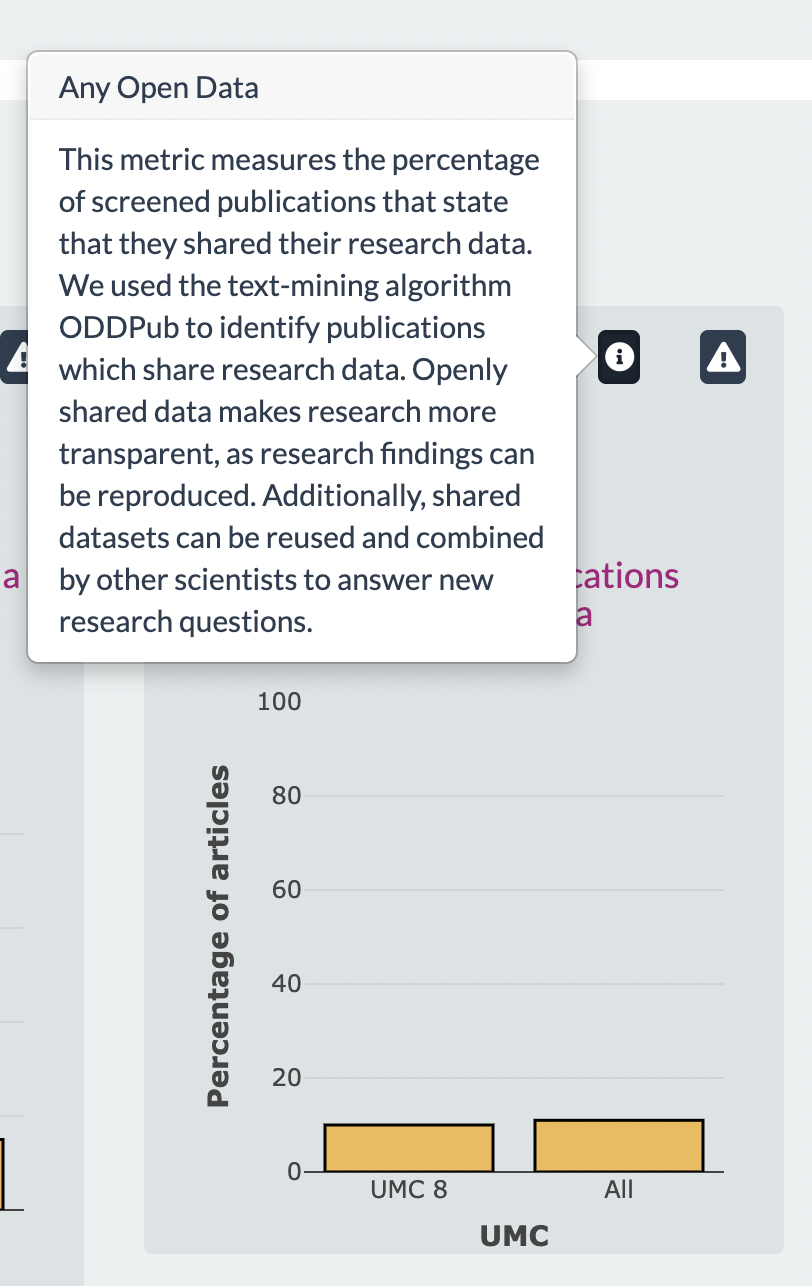

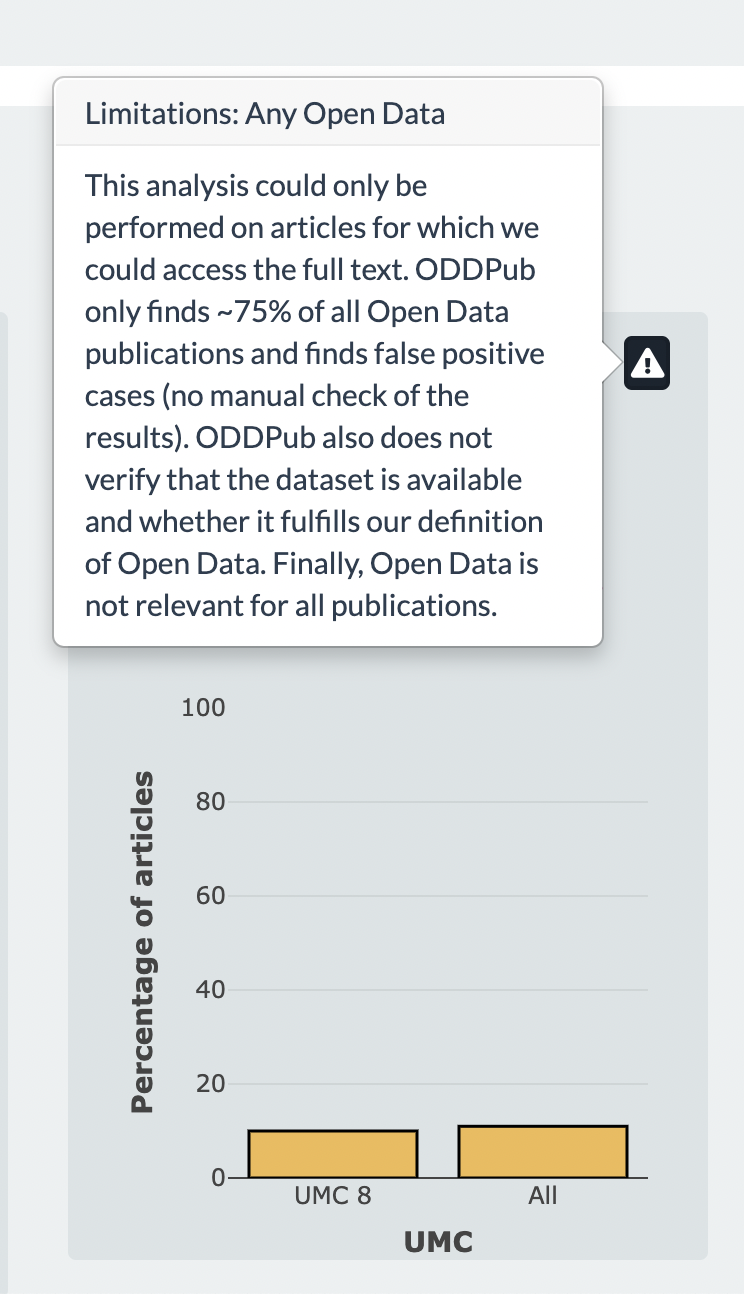

Supplement: S2 File — See also tutorial: https://www.youtube.com/watch?v=VDdljq5zI9E. (DOCX) [file pone.0269492.s002.docx]
