# Supplementary material for: Stakeholders’ views on an institutional dashboard with metrics for responsible research
Source: PLoS One. 2022 Jun 24;17(6):e0269492. doi: 10.1371/journal.pone.0269492 (PMC9231768; doi:10.1371/journal.pone.0269492)
Supplement: S3 File — Please note: Italics are alternative phrasings. (DOCX) [file pone.0269492.s003.docx]

**Supporting information 3.** Topic guide

# Topic guide -- SWOTs of Dashboard approach.

## Introduction interview

1. Open interview [“Thank you for participating in the interview”]

2.  Notify about recording [“I would like to emphasize that we record this interview, we will then transcribe the interview anonymously and after that the recording will be safely deleted.”]

3. Explanation of what will happen with the results [“The results will be used in a scientific paper, where we will illustrate our findings with quotes from the interview. These quotes are anonymized, but you might be able to recognise your own citations. However, nobody else should be able to.”]

4. Check informed consent [“Thank you for signing the informed consent form. I would like to repeat that participation is voluntary, that you can withdraw at any time without suffering any disadvantage. Do you have any questions before I start the recording?”]

5. Introduce yourself and themselves (name, position, institute) [“My name is TH and I work as postdoctoral researcher for QUEST that strives to increase the value of biomedical research at BIH and beyond. Could you perhaps introduce yourself?”]

6. Introduction project/goal interview: “We invited you to take part in these interviews that are part of the BRAVO project. The overarching goal of the BRAVO project is to increase the application of practices for robust and useful research across German University Medical Centers (UMCs). To realize this overarching goal, we are developing a dashboard that would allow UMCs to visualize the adoption of these responsible research practices by means of ‘metrics’ that relate to open science, timely reporting of clinical trials and robustness in animal research. The dashboard ‘dummy’ that you have been sent and are seeing now is our proof-of-principle version thereof. The goal of the dashboard is to provide institutions and other interested parties with a baseline indicator of the degree to which several practices for robust and open science are being performed at one specific institution. We invited you because we are very interested in your views on this dashboard and the metrics it includes. The goal of the interviews is for us to learn about the strengths and weaknesses of this dashboard and the metrics it includes, and to identify potential improvements. Do you have any other questions before we start?”

## Core interview: SWOTs of dashboard

**‘Grandtour’ question:**
What is your view on a dashboard using these novel metrics?

**Deepening questions:**

What would you consider the strengths and weaknesses of such a dashboard?

*What do you consider possible pitfalls of such a dashboard?*

*What is the added value of such a dashboard?*

*Based on the dashboard, do you feel you have a good understanding of the*  *limitations of metrics displayed?*

How would you, in your institution, use such a dashboard? – intended to yield ‘threats/opportunities’

*What would be, in your opinion, incorrect usage of such a dashboard?*

*What would/could you use such a dashboard for?*

*How should university leadership use such a dashboard?*

*What would be the possible (positive/negative) consequences of using this dashboard?*

*How could the information in this dashboard be abused, or what would be ‘gaming’ the information in this dashboard?*

*One foreseeable usage of this dashboard is that it could be used for benchmarking institutions, what are your thoughts regarding benchmarking?*

Which metric would you find most informative to support decision making at your institution? – intended to ‘zoom in’ on the SWOTs of the metric for the interviewee’s particular institute

* *How could this metric play a role?*

Which metrics do you believe should receive most attention and why? – if time is left to get insight into which metrics people are interested in

## Refinement and uptake questions

How should this dashboard be refined to promote uptake among UMC leadership?

*What suggestions do you have to further refine this dashboard?*

*Which feature of the dashboard should be improved (and how/why?)?*

*How could we optimise the dashboard to increase the chances of uptake among UMC leadership?*

**Snowballing** (optional)

With which other experts in your network should we talk to in order to better understand the situation?

## Ending the interview

Is there anything else you’d like to say?

Can I contact you in case I need any additional information or if something is unclear?

Can we send you a summary of this interview with the option to comment or send corrections? (Member-checking)

Close the interview and thank the interviewee for their participation.
