# Supplementary material for: Stakeholders’ views on an institutional dashboard with metrics for responsible research
Source: PLoS One. 2022 Jun 24;17(6):e0269492. doi: 10.1371/journal.pone.0269492 (PMC9231768; doi:10.1371/journal.pone.0269492)
Supplement: S4 File — Note: This code system consists of three levels. The SWOTs in bold (e.g., “Strengths”, highest level), the themes in italics (e.g., “data and behavior driven” medium level–different topics that could be interpreted as SWOTs), and subcodes in regular font (e.g., “pick a core set of metrics”, lowest level–subcomponents of a theme). (DOCX) [file pone.0269492.s004.docx]

Supporting information 4. Code Tree

*Note:* This code system consists of three levels. The SWOTs in bold (e.g., “Strengths”, highest level), the themes in *italics* (e.g., “data and behavior driven” medium level – different topics that could be interpreted as SWOTs), and subcodes in regular font (e.g., “pick a core set of metrics”, lowest level – subcomponents of a theme).

| **Code System** |
| --- |
| **Strengths** |
| *Seeing where you stand* |
| data and behavior driven |
| creating a baseline |
| self-evaluation |
| *Novel and relevant* |
| innovative and discipline-specific |
| alternative to current indicators |
| novel and timely |
| *Clear presentation* |
| percentages and absolute numbers |
| interactive features |
| **Weaknesses** |
| *Lack of justification for metrics included* |
| not properly embedded in the policy landscape |
| infrastructure needs to be in place first |
| metrics do not represent robustness |
| lack of reasoning behind the metrics |
| *Methods and conceptualization difficult to understand* |
| additional layer of data |
| unclear denominator |
| not all data sources covered |
| data sources and missingness not covered |
| metrics badly explained and operationalized |
| lack of consensus about terminology |
| qualifiers unclear (e.g., “Any…”) |
| *Possibly outdated* |
| attention for Open Science increased |
| **Opportunities** |
| *Initiating change* |
| fuel discussion how to improve |
| more holistic evaluation |
| add information how to induce change |
| induce collaboration or healthy competition |
| *Benchmarking over time* |
| benchmarking against yourself |
| tracking progress |
| *Internal usage only* |
| roll-out only after some delay |
| institutions choose what to make public |
| only the UMC leadership |
| *Tailoring dashboard* |
| allow institutions to comment/correct the numbers |
| more fine-grained indicators |
| pick and choose indicators relevant indicators |
| center level or research field level |
| *Complementing the dashboard with other indicators* |
| Open Science |
| diversity |
| preprints |
| General indicators |
| diversity |
| societal value and uptake of research |
| animal research |
| choice of model system |
| preregistration of animal research |
| clinical research |
| number of trials |
| patient engagement |
| turn dashboard into repository |
| *Communicating metrics’ performance* |
| sensitivity and specificity |
| enrich information conveyed, e.g. with error bars |
| information on dashboard updates |
| **Threats** |
| *Putting institutions in a bad light* |
| correct the records |
| not to be blamed for lack of infrastructure |
| *Incorrect interpretation* |
| dynamic information |
| choice of comparator |
| numbers taken at face value |
| lack of context |
| *Gaming metrics* |
| numeric indicators always wrong |
| manipulating information |
| Goodhart’s law |
